# Supplementary material for: Simultaneous Quantification of Bisphenol-A and 4-Tert-Octylphenol in the Live Aquaculture Feed Artemia franciscana and in Its Culture Medium Using HPLC-DAD
Source: Methods Protoc. 2022 May 1;5(3):38. doi: 10.3390/mps5030038 (PMC9149995; doi:10.3390/mps5030038)
Supplement: Supplementary file 1 [file mps-05-00038-s001.zip › mps-1690169-supplementary.pdf]

Supplement

A

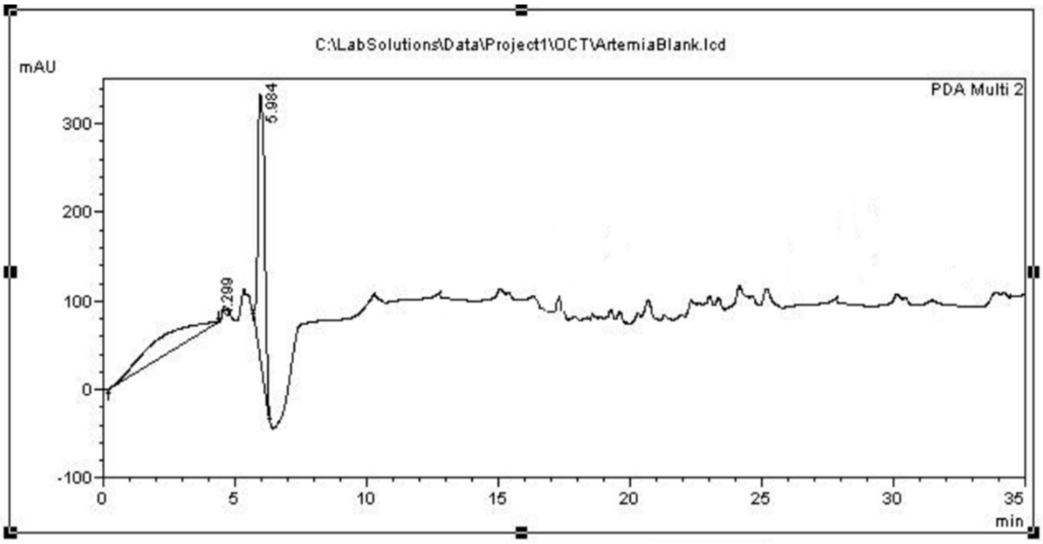

B

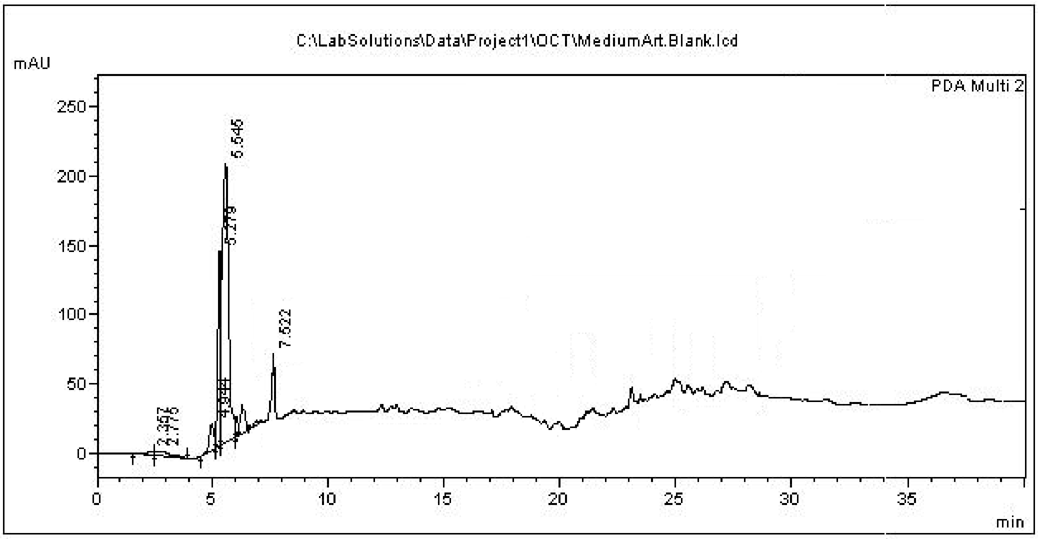

Figure S1. HPLC chromatograms of blank Artemia tissue (A) and culture medium (B) samples.

A

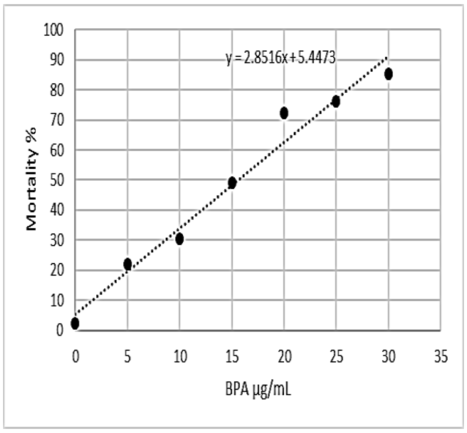

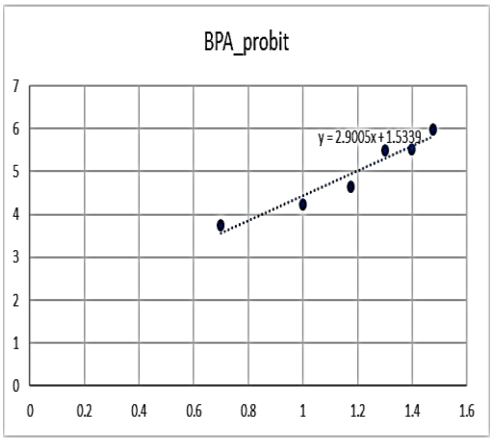

LC50=15.62 **LC50=15.66**

B

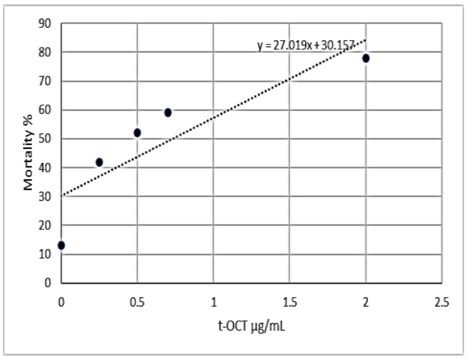

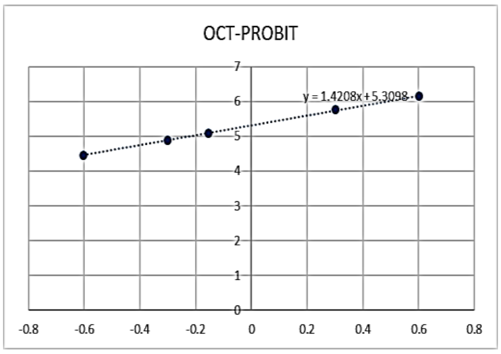

LC50=0.73 **LC50=0.61**

Figure S2. Linear Regression analysis for the determination of LC_{50} values of BPA and t-OCT

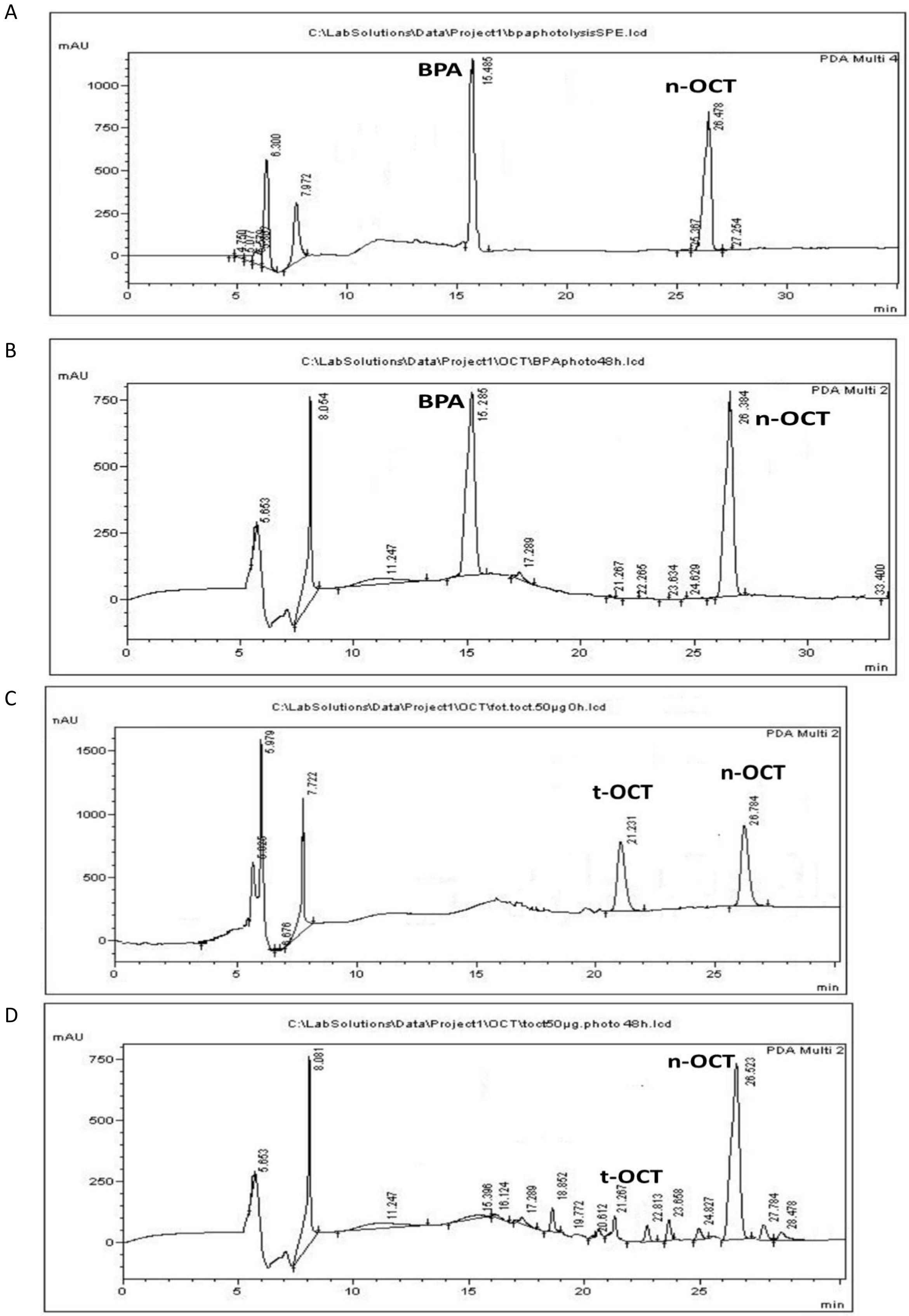

Figure S3. Photodegradation of BPA and t-OCT. Chromatograms of medium with (A)BPA at 0h, (B) BPA at 48h, (C) t-OCT at 0h, (D) t-OCT at 48h
